# Helicoidally Arranged Polyacrylonitrile Fiber-Reinforced Strong and Impact-Resistant Thin Polyvinyl Alcohol Film Enabled by Electrospinning-Based Additive Manufacturing

**DOI:** 10.3390/polym12102376

**Published:** 2020-10-15

**Authors:** Rahul Sahay, Komal Agarwal, Anbazhagan Subramani, Nagarajan Raghavan, Arief S. Budiman, Avinash Baji

**Affiliations:** 1 Engineering Product Development, Singapore University of Technology and Design, Singapore 487372, Singapore; rahul@sutd.edu.sg (R.S.); komal_agarwal@mymail.sutd.edu.sg (K.A.); M130148@e.ntu.edu.sg (A.S.); nagarajan@sutd.edu.sg (N.R.); 2 Industrial Engineering Department, BINUS Graduate Program—Master of Industrial Engineering, Bina Nusantara University, Jakarta 11480, Indonesia; 3 Department of Engineering, La Trobe University, Bundoora, VIC 3086, Australia

**Keywords:** impact resistance, bioinspired, helicoidal structure, electrospinning

## Abstract

In this study, we demonstrate the use of parallel plate far field electrospinning (pp-FFES) based manufacturing system for the fabrication of polyacrylonitrile (PAN) fiber reinforced polyvinyl alcohol (PVA) strong polymer thin films (PVA SPTF). Parallel plate far field electrospinning (also known as the gap electrospinning) is generally used to produce uniaxially aligned fibers between the two parallel collector plates. In the first step, a disc containing PVA/H_2_O solution/bath (matrix material) was placed in between the two parallel plate collectors. Next, a layer of uniaxially aligned sub-micron PAN fibers (filler material) produced by pp-FFES was directly collected/embedded in the PVA/H_2_O solution by bringing the fibers in contact with the matrix. Next, the disc containing the matrix solution was rotated at 45° angular offset and then the next layer of the uniaxial fibers was collected/stacked on top of the previous layer with now 45° rotation between the two layers. This process was continued progressively by stacking the layers of uniaxially aligned arrays of fibers at 45° angular offsets, until a periodic pattern was achieved. In total, 13 such layers were laid within the matrix solution to make a helicoidal geometry with three pitches. The results demonstrate that embedding the helicoidal PAN fibers within the PVA enables efficient load transfer during high rate loading such as impact. The fabricated PVA strong polymer thin films with helicoidally arranged PAN fiber reinforcement (PVA SPTF-HA) show specific tensile strength 5 MPa·cm^3^·g^−1^ and can sustain specific impact energy (8 ± 0.9) mJ·cm^3^·g^−1^, which is superior to that of the pure PVA thin film (PVA TF) and PVA SPTF with randomly oriented PAN fiber reinforcement (PVA SPTF-RO). The novel fabrication methodology enables the further capability to produce even further smaller fibers (sub-micron down to even nanometer scales) and by the virtue of its layer-by-layer processing (in the manner of an additive manufacturing methodology) allowing further modulation of interfacial and inter-fiber adherence with the matrix materials. These parameters allow greater control and tunability of impact performances of the synthetic materials for various applications from army combat wear to sports and biomedical/wearable applications.

## 1. Introduction

Research has been ongoing to study the mechanical properties of micron/sub-micron thin polymer films due to their widespread applications such as protective and functional coatings, non-fouling surfaces, microfluidics, sensors, lubrication and friction modification [1,2,3,4]. Typically, the successful long-term performance and reliability of such thin films are governed by their mechanical properties [5,6,7,8]. Common strategies used for the fabrication of strong polymer thin films (SPTFs) include the addition of inorganic material such as carbon tubes, carbon fibers, 2D materials such as graphene oxide and metallic/metal oxide particles in the thin polymeric films [5,7,8]. Blending with strong polymeric material is also used for the fabrication of SPTFs [9,10]. Nevertheless, these methodologies may result in an increase in the specific weight, incompatibility with inorganic material, anisotropic mechanical properties due to nonuniform distribution of the filler material, void formations and/or high cost of the resultant composite [11], reducing their strength. Therefore, nano/sub-micron fibers are being used as fillers due to their large surface area per unit volume to be completely enclosed by the matrix materials thus avoiding the formation of voids or stress concentration zones. Further, it is now known that structural hierarchical arrangement of nano/sub-micron fibers in thin films improves its tensile and impact properties [12,13,14].

Therefore, researchers are taking inspiration from the multiscale (nano/sub-micron) hierarchical architectures found in natural structural materials, such as in mantis shrimps’ dactyl club, nacre, lobster claw and butterfly wing, to design next-generation of strong and/or tough materials [15,16,17]. For instance, natural structural material found in mantis shrimps’ dactyl club consists of helicoidally arranged chitin fiber mineralized by hydroxyapatite [18]. This helicoidal architecture enables mantis shrimp to sustain high impact energy while smashing its prey. The helicoidal arrangement of the chitin fibers embedded in hydroxyapatite allows deflection of cracks and dissipation of the impact energy along distinct planes and directions to avoid catastrophic failure during impact [19]. The remarkable mechanical properties such as high strength (over 100 MPa) [20], impact resistance (33 J·m^−1^ for 10 ball drop impact) [21,22] and toughness of such natural structural materials strongly depend on the synergistic effects from architecture interfacial interactions as well as their multiscale hierarchical design [23,24,25,26]. Such geometrical inspiration from the natural fibrous structure can be employed to improve the strength/toughness of microscopic polymeric thin films. In this article, such helicoidal geometry has been taken as an inspiration to fabricate helicoidally aligned sub-micron sized fibers to reinforce the SPTFs.

Techniques such as conventional prepreg/ply stacking and 3D printing have been used in the past for mimicking the helicoidal structures [27,28,29,30,31]. However, conventional prepreg stacking usually produces macroscopic sample and 3D printing can only achieve microscopic fiber with the limitation of available constituent materials. In addition, the materials with smaller scale (sub-micron down to nanometer) structural features have received wide attention in the past two decades [32,33,34]. Nanowires [35], nanopillars [36] or bulk materials with nanoscale grain structure [37] or nanolayers [38,39,40,41] have shown excellent mechanical properties, including the combination of strength and toughness. The desirable properties of the molecular orientation and mechanical properties (strength, toughness and impact-resistance) occurring due to the smaller size effect have motivated fabrication of the structural geometries in the sub-micron/nano level [32,36,42]. Therefore, electrospinning has been used in this article for its capability of producing sub-micron to nanometer-scale fibers [43,44], for having the ability to use various types of constituent materials (polymers, metal particles, carbon nanotubes (CNTs), cellulose nanocrystals (CNCs), ceramics, metal powders and nanoparticles) [42] and for allowing tunability of the electrospinning parameters (such as voltage, solution viscosity, fiber size and nozzle to collector distance).

The objective of this article is to perform extended studies towards achieving nano-sized helicoidal architecture using a single-step fabrication method. In this study, a new parallel plate far field electrospinning (pp-FFES) based methodology was used for the first time to fabricate complex 3D helicoidal geometrical composites within a single fabrication step. During a typical electrospinning process, electrical forces are applied to a polymer solution dispensing out of a nozzle to produce randomly oriented 3D fibrous membrane. The extruding polymer solution experiences bending and whipping instabilities due to the applied electrical forces and lays down fibers in random fashion on top of the collector, whereas parallel plate electrospinning setup is a common technique used to generate uniaxially aligned fibrous layers, by reducing the bending and whipping instabilities by placing sharp edged parallel plates within the electrical field [45,46] (see Figure A6). However, to the best of our knowledge, pp-FFES has not been used for generating complex hierarchical geometrical network of nano fibers so far, let alone the helicoidal architecture. In this study, a matrix solution/bath (PVA/H_2_O) was placed in between the two parallel plate collectors, such that, as soon as the fibers formed between the two-plates, the fibers were brought in contact with the matrix solution. Initially, a single layer of uniaxial arrays of PAN fiber was obtained between the two parallel plates, which was then directly collected into the matrix solution by bringing fibers in contact with the PVA/H_2_O solution in the disc. The extra fibers hanging outside the periphery of the disc were trimmed off neatly. Then, the disc containing the matrix solution was rotated at 45° angular offset, and, again, the next layer of the uniaxial arrays of PAN fibers was collected/stacked on top of the previous layer with now 45° angular offset between the two layers. This process was continued with progressive 45° angular offsets between the layers until a periodic pattern was achieved. In total, 13 such layers were laid within the matrix solution. This layer-by-layer process is indeed vital for completely embedding PAN fibers in the PVA/H_2_O solution and providing better surface interaction between the fibers and the matrix material, as compared to the conventional method of post impregnation of fibers in the matrix materials. This methodology is thus superior to the state of the art where pre-prepared helicoidal fiber structures are embedded in the matrix material, which may not allow complete surrounding of filler fibers by matrix [47]. Moreover, the current state of the art used for fabrication of helicoidal geometry in the composites is limited by constituent materials, pre-prepared fillers, prepregs and the fiber size. This study eliminates these limitations for generation of the sub-micron to nano sized helicoidal geometrical architecture. The authors propose that the smaller fibers (sub-micron to nanometer scale) in the helicoidal composites could enable further enhancement in the impact performance (measured by its ability to protect the glass coverslip lying underneath it from fracturing) as the more interfacial surfaces (between fibers as well as layers) per unit volume of the materials would allow very efficient dissipation of energy, thus greatly enhancing the impact resistance of the materials. However, electrospinning methodologies are relatively a newer platform for fabrication of hierarchical complex structural geometries in nano range. Hence, furthermore advancements in electrospinning techniques are desired to obtain even more dense uniaxial fibers, even better alignment and uniformity for fabricating superior helicoidal composites in the future.

The relationship between architecture, interfacial interactions and the mechanical properties of samples were systematically analyzed in this study. The morphology, structure and composition of the samples were investigated through scanning electron microscopy (SEM) and Fourier-transform infrared spectroscopy (FTIR). Thermogravimetric analysis (TGA) and differential scanning calorimetry (DSC) were used to analyze the thermal properties of the samples. The mechanical properties of the samples were studied through uniaxial tensile tests and free-falling steel ball impact test [12,48].

## 2. Material and Methods

### 2.1. Materials

Polyvinyl alcohol (PVA, *Mw* = 130,000 Da), polyacrylonitrile (PAN, *Mw* = 154,000 Da) and dimethyl formaldehyde (DMF) were purchased from Merck & Co. (Kenilworth, NJ, USA) and used without any further purification. The syringe and needles used during electrospinning were purchased from Terumo, Tokyo, Japan.

Polyvinyl alcohol was used as a matrix material in this study due to its ability to dissolve in water (allowing to control the viscosity of the matrix solution) and the ability to form thin films. PVA also serves a wide variety of applications including wound dressing, biomedical and food packaging [49,50,51,52]. Nevertheless, PVA thin films suffer from low tensile strength (∼10 MPa) [53]. Various strategies are being employed to improve the mechanical properties of PVA thin films such as the addition of starch/critic acid [54], nanofibrillated cellulose [55] and halloysite nanotubes [52]. However, these filler materials were randomly distributed in the thin films. The size of the thin film is also governed by the size of the fibers or particles added to improve its mechanical properties. Therefore, helicoidally arranged polyacrylonitrile sub-microfibers were used for reinforcing PVA to improve its mechanical properties [48,56]. Further, polyacrylonitrile filler sub-microfibers were used for reinforcing PVA due to their high Young’s modulus (∼3.1 GPa) [57] and compatibility with PVA. PAN and PVA interact through hydrogen bonding [58].

### 2.2. Methods

**Figure 1:**
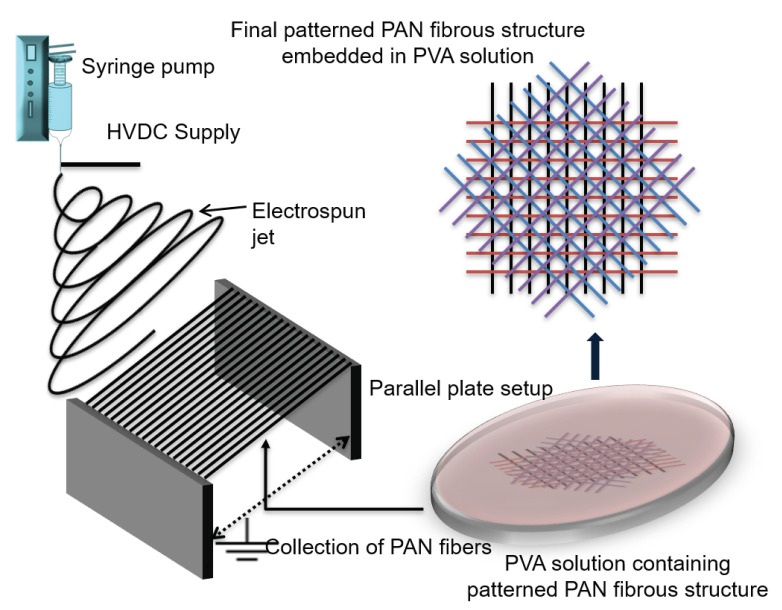
Fabrication of polyvinyl alcohol (PVA) strong polymer thin films with helicoidally arranged polyacrylonitrile (PAN) fiber reinforcement (i.e., PVA SPTF-HA). Uniaxial arrays of PAN fibers generated using parallel plate far field electrospinning (pp-FFES) were embedded directly into a circular disc containing PVA/H_2_O solution/bath by bringing them in contact with solution in the disc. The disc was then rotated progressively at 45° angular offsets to stack three pitches of helix to form PVA SPTF-HA.

**Figure 2:**
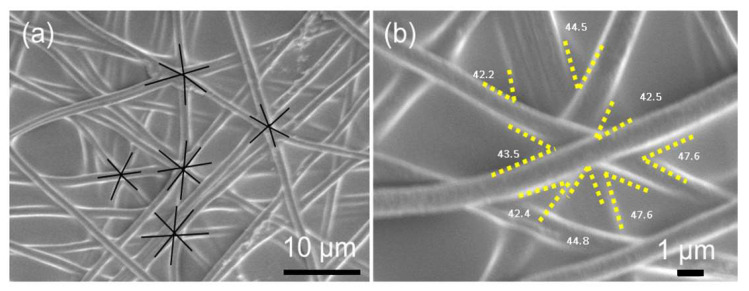
(**a**) SEM image depicting the morphology of fabricated PVA SPTF-HA; and (**b**) magnified image of PVA SPTF-HA with helicoidally arranged PAN fibers. Here, 13 helicoidally arranged PAN fibrous layers with 45° ± 3° offset angle are embedded layer by layer in 5 wt% PVA/H_2_O solution and later dried to fabricate PVA SPTFs.

**Figure A1:**
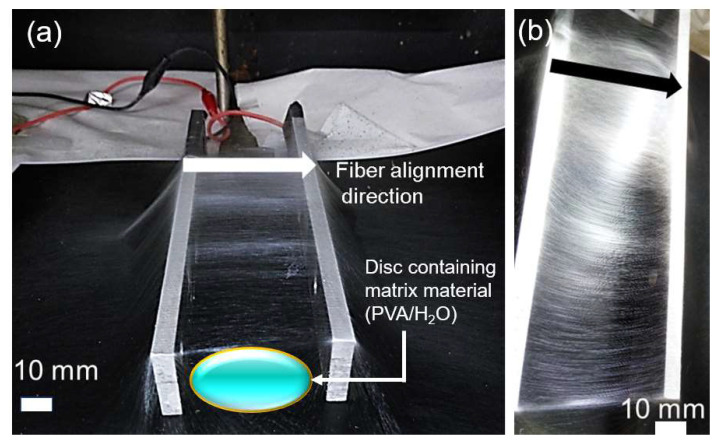
(**a**) Parallel plate far field electrospinning methodology used for the fabrication of uniaxially aligned PAN fibers. (**b**) These uniaxially aligned fibers were then collected in the PVA solution. The process is repeated 13 times to generate PVA SPTF-HA. The arrow in the figure shows the fiber alignment direction.

**Figure A2:**
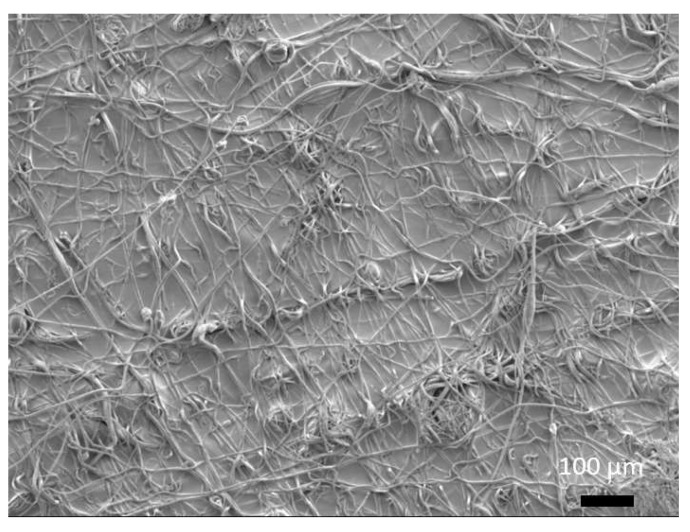
PVA SPTF-RO prepared using conventional electrospinning methodology.

Typically, fabricated PVA strong polymer thin film (PVA SPTF) consisted of PAN fibers embedded in PVA and two types of samples were made: (i) helicoidally arranged PAN fibers (diameter = (0.93 ± 0.1) μm) embedded in PVA thin film (PVA SPTF-HA) (see Figure 2 and Figure A4); and (ii) randomly oriented PAN fibers (diameter = (3.31 ± 1) μm) embedded in PVA thin film (PVA SPTF-RO) (see Figure A2 and Figure A5 in Appendix A, respectively). Initially, 10 wt% PAN was dissolved in DMF using a hot-plate magnetic stirrer (Thermo Fisher Scientific, Waltham, MA, USA) at 60 °C. This solution was fed into the syringe for electrospinning (Nanospinner-24, Inovenso, Turkey). A custom-built parallel plate fiber collection setup was used for the arrangement of uniaxial arrays of PAN fibers in a hierarchical helicoidal geometry with 45° angular offsets directly within the matrix solution (see Figure 1). A digital syringe pump (Premier Solution, Singapore) was used to extrude the PAN solution at a flow rate of 3 mL·h^−1^. The electric field used during electrospinning was 1 kV·cm^−1^ between the spinneret and the parallel plate collectors (see Figure A1, Appendix A). The distance between the spinneret to the parallel plate was 15 cm and the distance between the parallel plates was 6 cm. A disc of 50 mm diameter and 3 mm depth filled with PVA/H_2_O matrix solution was placed in between the two plates such that the uniaxial fibers get directly deposited/embedded in the matrix solution by bringing it in contact with the solution in the disc. Once an array of uniaxial PAN fibers were collected in the matrix bath, the disc was manually rotated progressively at 45° angular rotations to align the uniaxial arrays of the fibers in a helicoidal stack. A periodic pattern of continuous 45° offsets (i.e., 0°, 45°, 90°, 135°, 180°, 225°, 270°, 315°, 360/0°, until the next 180°) from the previously deposited layers were followed until three pitches of helicoidal geometry were obtained (one pitch means half a turn of the helix, i.e., 0° to 180°, then 225° to 360/0° and, lastly, 45° to 180°). Thus, 13 layers of uniaxial arrays of PAN fibers were stacked one above the other with 45° angular offsets (see Figure 2). After electrospinning each layer of uniaxial fibers inside the disc with the matrix solution, the excess fibers hanging between the disc and the edge of the parallel plate were carefully trimmed off using a pair of scissors. This was done to detach the fibers from the parallel plates while avoiding any stretching, twisting and misalignment when rotating the disc at 45° offset to collect the next set of uniaxial layers.

Various weight% (1 to 5 wt%) of PVA/H_2_O solutions were prepared to embed aligned electrospun fibers. Nevertheless, only 5 wt% PVA/H_2_O solution was able to form uniform thin film with PAN fibrous layers embedded in it. Hence, 5 wt% PVA/H_2_O solution was chosen in the fabrication of all the samples for all the experiments in this article. The authors believe that layer by layer insertion of uniaxially aligned PAN fibers into the PVA bath/solution would provide better surface interaction between the impregnating solution and the PAN fibers as compared to post impregnation of fully formed helicoidally arranged fibrous structure. Later, the disc containing the PVA/H_2_O soaked PAN films was kept undisturbed at the room temperature for drying (by the simple evaporation process) for 3 days to remove water and result in the formation of PVA strong polymer thin films with helicoidally arranged PAN fiber reinforcement (PVA SPTF-HA). The randomly oriented PAN fiber reinforced PVA strong polymer thin films (PVA SPTF-RO) were fabricated using typical random fibrous film prepared by conventional electrospinning setup, while following the above electrospinning parameters and without using the parallel plates. The time (for electrospinning) required for the fabrication of random PAN fibrous film for PVA SPTF-RO was same as for the fabrication of 13 uniaxially aligned PAN fibrous layers for PVA SPTF-HA so as to keep the amount of PAN same for both PVA SPTF-RO and PVA SPTF-HA. The fiber weight percentage in both the type of composites was kept ∼1 wt%.

A pure/neat PVA thin film (PVA TF) was also fabricated by pouring the 5 wt% PVA/H_2_O solution on a flat circular disk of dimension 50 mm diameter and 3 mm depth and allowing it to dry for 3 days. Once the PVA film dried due to the evaporation process, it was peeled from the disc and used for experiments for comparison with PVA SPTFs. Next, the pure/neat PAN fibers were collected on an aluminum foil in random orientation, using the same PAN solution and electrospinning parameters as above, but without the parallel plates or the disc containing the matrix material. In this case, the PAN fibers were electrospun for 30 mins to obtain a film thick enough to easily peel it away from the foil. These PAN fibers were then dried in an oven at 60 °C for a day to obtain a dried electrospun neat PAN thin film (PAN TF). This PAN TF sample was later used for determining its chemical and thermal properties for comparison with PVA SPTFs.

### 2.3. Characterization

A field-emission scanning electron microscope (FESEM) (JEOL (JSM—6700F), Tokyo, Japan) was used to observe the morphology and arrangement of the PAN fibers in PVA SPTF samples. The thickness of the samples was measured from their cross-sectional images obtained through FESEM. The density of the samples was calculated with and without the fibrous reinforcement from the weight and the volume measurements of the samples. The density of samples was calculated by dividing the total mass of the fabricated sample by the volume of the sample. Fourier transform infrared spectroscopy (FTIR, Bruker Alpha spectrometer, Billerica, MA, USA) were recorded at a resolution of 4 cm^−1^ at room temperature. Thermogravimetric analysis (TGA, TA instruments (Q50), New Castle, DE, USA) was used to document the thermal stability of the samples at a heating rate of 10 °C·min^−1^. Differential scanning calorimetry (DSC, TA instruments (Q20), New Castle, DE, USA) tests were performed from −60 °C to 250 °C with a ramp rate of 3 °C·min^−1^ in a nitrogen atmosphere (flow rate 40 mL·min^−1^).

**Figure A3:**
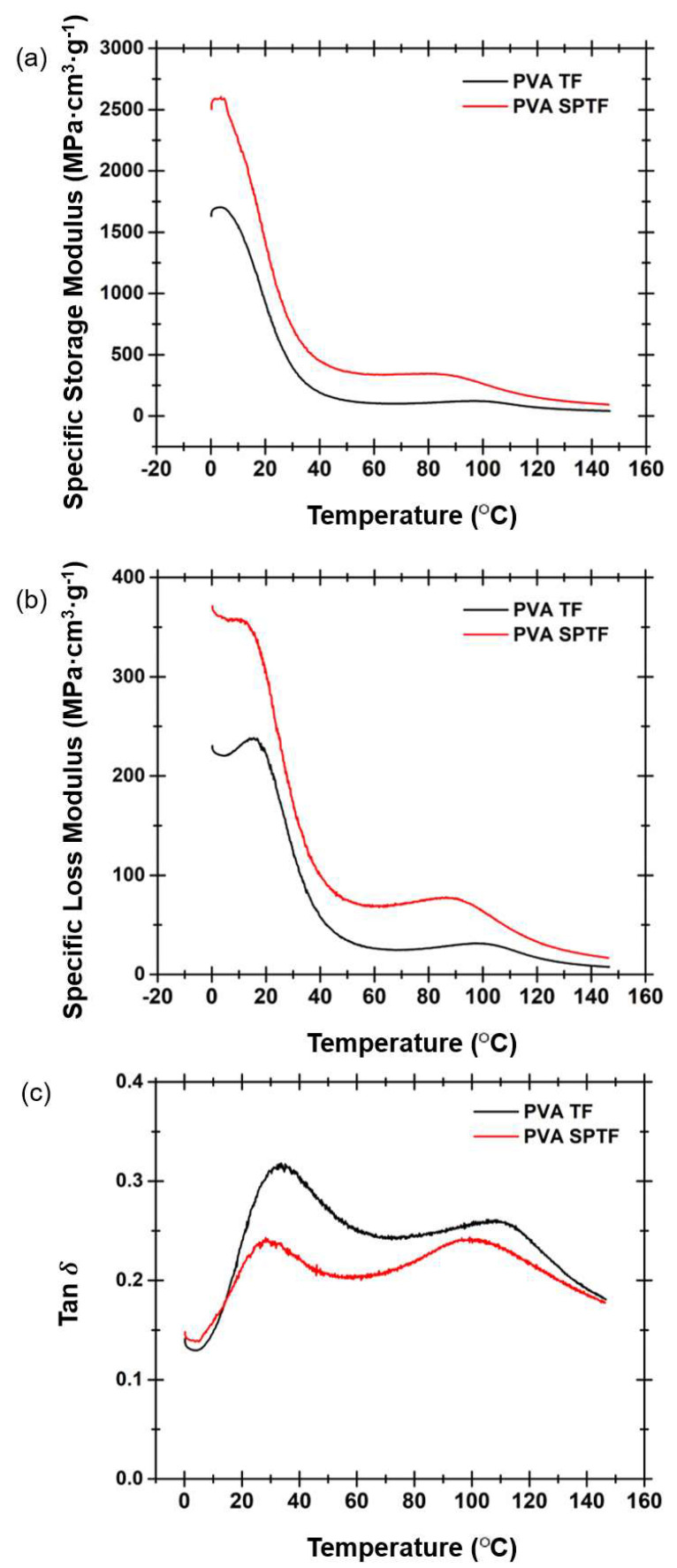
Specific viscoelastic properties of the PVA SPTF and PVA TF- (**a**) storage modulus; (**b**) loss modulus; (**c**) tan delta.

The mechanical properties of the samples were investigated using a tensile testing machine (MTS Criterion Model 43, MTS Systems Corporation, Eden Prairie, MN, USA). The typical sample dimensions used for testing were thickness ∼50 μm, width 5 mm and length 25 mm. The tensile tests were performed at a constant strain rate of 1 mm·min^−1^ and the clamp pressure was maintained at 25*psi*. Neat PVA thin film (PVA TF), helicoidally arranged PAN fiber reinforced PVA SPTF (PVA SPTF-HA) and randomly oriented PAN fiber reinforced PVA SPTF (PVA SPTF-RO) were tested for their mechanical properties. The weight percentage of the PAN fibers in the composites was kept around ∼1 wt%. The tests were performed for at least 5 specimens to check the repeatability of the results. A customized setup was used to determine the impact-resistance of the samples. The impact test was carried out using a free-falling steel ball to impact the sample with a given potential energy [12,48], and the performance of the samples was determined by the ability of the samples to protect a glass coverslip placed underneath the samples. The glass coverslip (14 mm diameter, 0.16 mm thickness) was made of borosilicate glass (*D*263, deckglaser, Trade 21, Singapore). The samples were adhered to the glass coverslip at the diagonal edge points so as to eliminate the effects of glue on the on the measurements and avoid the sample from moving from its place upon impact. The potential energy of the steel ball can be varied either by changing the mass or impact height of the steel balls. The mass of the steel ball used for the measurement was 0.51 g and its diameter was 4 mm, whereas the height from which steel ball is dropped was varied. The height from which the steel ball is dropped was initially increased with an increment of 5 cm. The initial experiments enabled to obtain initial tentative height required for the fracture of the glass coverslip with/without the samples. Later, the height in the vicinity of already obtained initial tentative fracture height was varied in the increments of 1 cm to obtain the exact value of height required to fracture the glass coverslip with/without the samples. Later, the potential energy of the steel ball required to fracture the glass coverslip with/without the samples were documented. All the samples used for impact tests were ∼50 μm thick and had a cross-sectional area of 1.1 × 1.1 cm^2^. Dynamic mechanical analysis (DMA, DMA Q800, TA Instruments, New Castle, DE, USA) was used to understand the specific viscoelastic properties of the samples. The scanning temperature range was selected from 0 °C to 150 °C at a strain ramp rate of 1%·min^−1^ and 1 Hz frequency in the multi strain mode (see Appendix A and Figure A3).

## 3. Results and Discussion

### 3.1. Physical Characterization of PVA SPTF

**Figure A4:**
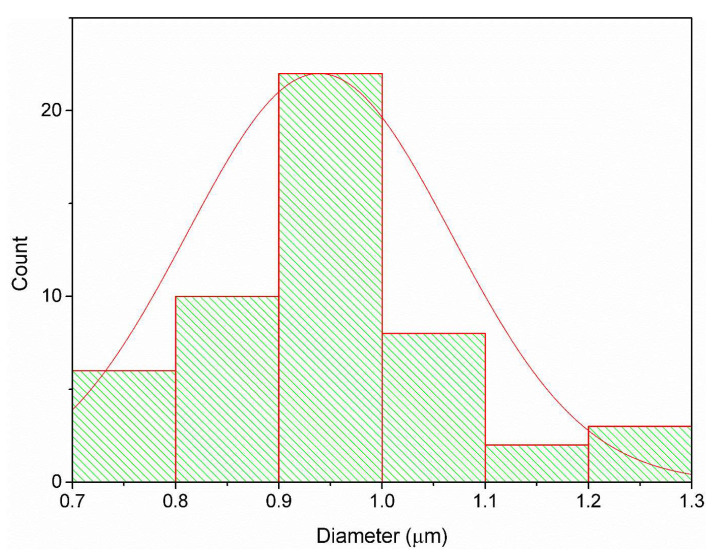
The diameter distribution of PVA SPTF-HA prepared through parallel plate far field electrospinning setup.

**Figure A5:**
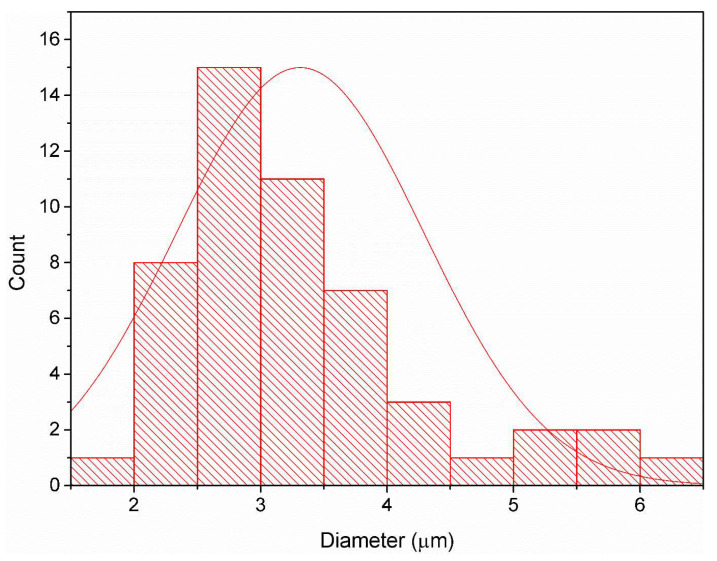
The diameter distribution of PVA SPTF-RO prepared using conventional electrospinning methodology.

**Figure A6:**
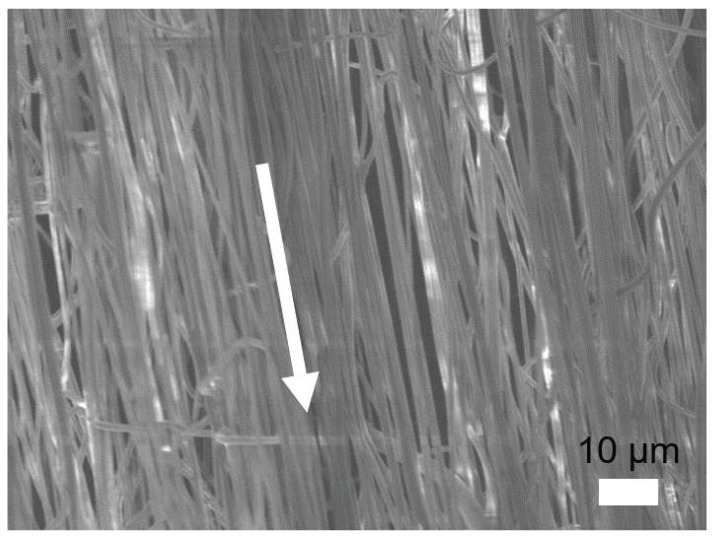
The SEM image of thick aligned PAN fibrous membrane obtained after electrospinning performed for an extended period of time using the parallel-plate setup shown in Figure A1. The white arrow shows the direction of fiber alignment.

First, the parallel-plate far field electrospinning was used for producing uniaxial arrays of PAN fibers. Then, these uniaxial PAN fibers were arranged helicoidally to obtain PVA SPTF-HA samples. Figure 2a,b shows the morphologies of the helicoidally arranged uniform and smooth PAN fibers (diameter = (0.93 ± 0.1) μm, see Figure A4, Appendix A) that were embedded within the PVA thin films (sample PVA SPTF-HA). The arrangement of layers consisting of aligned fibers one above the other at 45° angle within the PVA matrix is evident from these figures. Similar observations are also reported in the literature [12,48]. Due to the small size of the fibers, densely aligned fibers were not observed in the SEM images—similar to other reports in the literature, especially on a smaller range of fibers size [12]. It is significant that this novel technique has allowed the fabrication of helicoidally arranged fibers architectures with diameters of (0.93 ± 0.1) μm, while many other previous reports have conducted their studies with much larger fibers [59,60,61]. This technique would thus allow us to further investigate the small size effects (sub-micron and down to nanometer scales) of the fibers on the mechanical properties of the composites. Figure A2 (in Appendix A) shows the SEM image of the randomly oriented PAN fibers reinforced in PVA thin films (sample PVA SPTF-RO). This sample shows that the pp-FFES methodology gives better control in fiber orientation than compared to the electrospinning done without using parallel plate setup.

Next, the fabricated PVA SPTF samples are chemically, and thermally characterized and compared with the neat PVA thin film (PVA TF). These characterization techniques help in identifying the components in the samples as well as confirmation of the interfacial interaction between PAN and PVA. The good interfacial adhesion between the matrix and the fibers is expected to improve the mechanical deformation behavior of the PVA SPTF. The mechanical properties of PVA SPTF-HA samples are then characterized using uniaxial tensile and impact resistance experiments and compared with the PVA SPTF-RO and PVA TF, as discussed in the subsequent sections. The comparison of the mechanical properties helps in understanding the toughening and impact-resistant mechanisms of the hierarchical helicoidal arrangement of the fibers in the matrix, over the neat matrix and randomly oriented fiber reinforced thin films.

### 3.2. Chemical and Thermal Properties of PVA SPTF

#### 3.2.1. Fourier-Transform Infrared Spectroscopy (FTIR)

**Figure 3:**
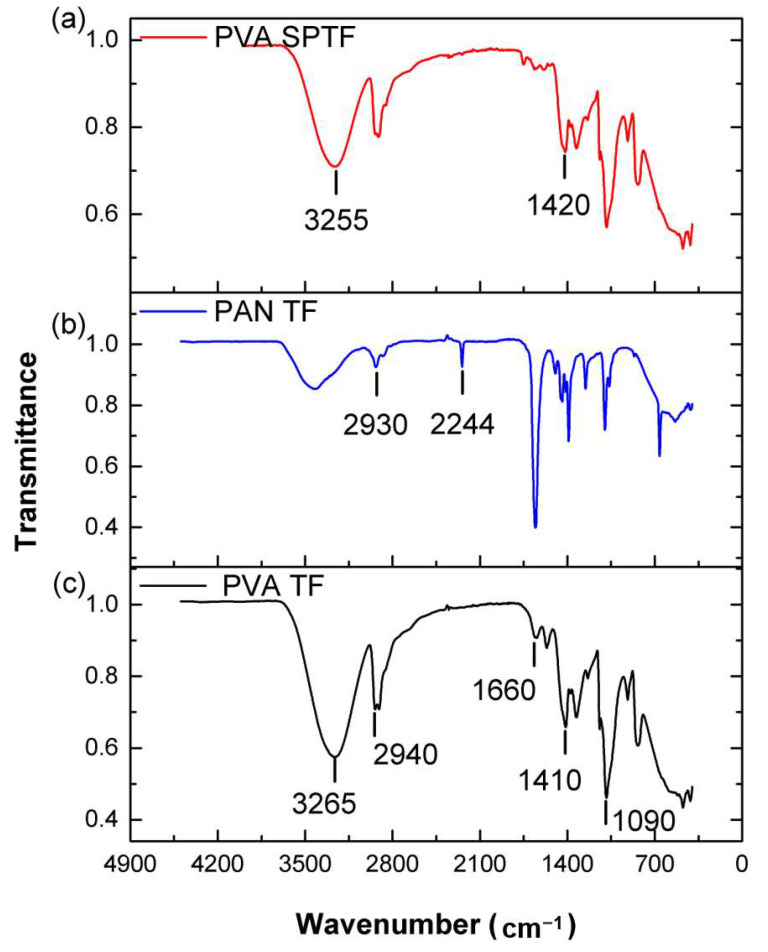
FTIR plots of: (**a**) PVA SPTF; (**b**) neat PAN thin film (PAN TF); and (**c**) PVA TF.

The FTIR scan of neat PVA shows the characteristic peaks of PVA associated with -OH- stretching and –OH– bending peaks that are recorded at 3265 and 1410 cm^−1^ (see Figure 3c). The asymmetric stretching of methylene group –CH_2_- is recorded at 2940 cm^−1^. The peaks at 1090 and 1660 cm^−1^ are associated with –C–O– and –C–C– bond stretching, respectively [62]. FTIR scan recorded for the PAN fibers demonstrate the characteristic peaks associated with PAN, which include stretching vibration peaks of –CH_2_- at 2930 cm^−1^. The peak at 2244 cm^−1^ corresponds to the stretching vibrations of –C≡N–. In the case of PVA SPTF, the characteristic peaks associated with –OH– stretching decreased with the addition of PAN in PVA SPTF, which is associated with hydrogen bonding [63]. Typically, stretch peaks redshift to lower wavenumber, whereas bending peaks blue shift to a higher wavenumber due to the presence of hydrogen bonding initiating groups. The –OH– stretching peak for PVA shifts to a lower wavenumber (3255 cm^−1^) and –OH– bending peaks shifted to a higher wavenumber (1420 cm^−1^) when PAN is incorporated into the composite. This is attributed to the formation of hydrogen bonds between the hydroxyls of PVA and the nitriles of PAN.

#### 3.2.2. Thermogravimetric Analysis (TGA)

**Figure 4:**
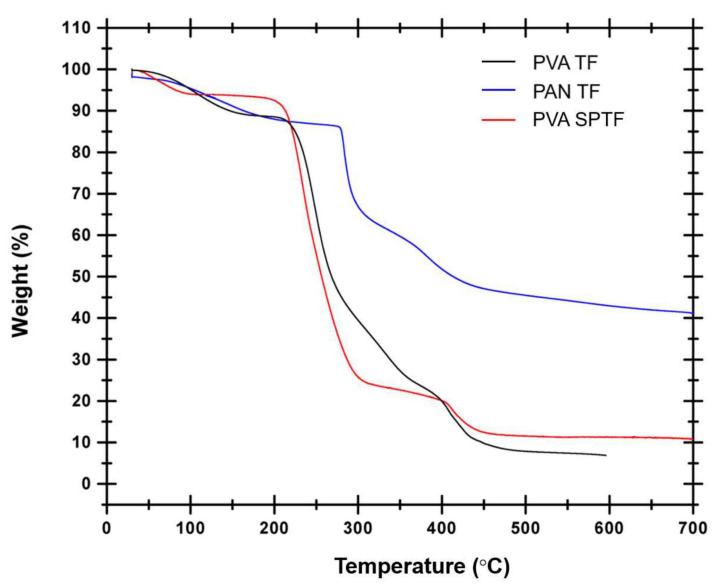
TGA thermograms of PVA SPTF, PAN TF and PVA TF.

Figure 4 shows the TGA curves of neat PAN, PVA and PVA SPTF. An initial weight loss of 11% is seen for the PAN sample between 25 and 183 °C, which is attributed to the loss of moisture as well as the solvent present in the sample. Later, the PAN sample lost 3% weight in the temperature range of 183–279 °C, which can be attributed to dehydrogenation reactions and release of volatile compounds [64]. Then, the PAN macromolecule started degrading with an increase in the applied temperature from 279 to 700 °C with the final weight loss of around 60% [65]. At 700 °C, it is observed that the PAN fibers get partially carbonized. Neat PVA depicts a weight loss of 13% from room temperature to 210 °C. This weight loss can be further divided into two temperature ranges: one from room temperature to 163 °C corresponding to the loss of moisture and the other for 163–210 °C corresponding to the condensation of the hydroxyl groups in the PVA TF [66]. Later, the degradation of PVA macromolecule commences at 210 °C and finishes at 450 °C with a reduction to 10% of its initial weight [62]. The final weight loss of the PVA thin film is 93 wt% at 595 °C [67].

Similarly, the PVA SPTF lost 9% weight during 25–210 °C. This weight loss can be further divided into two temperature ranges. The first temperature ranges from 25 to 108 °C corresponds to the loss of moisture, and the second from 108 to 210 °C corresponds to the condensation of the hydroxyl groups in the PVA SPTF. Further, the PVA SPTF show a higher degradation temperature 700 °C (9% residual weight) in comparison to the neat PVA film 595 °C (6% residual weight) due to the presence of PAN fibers.

#### 3.2.3. Differential Scanning Calorimetry (DSC)

**Figure 5:**
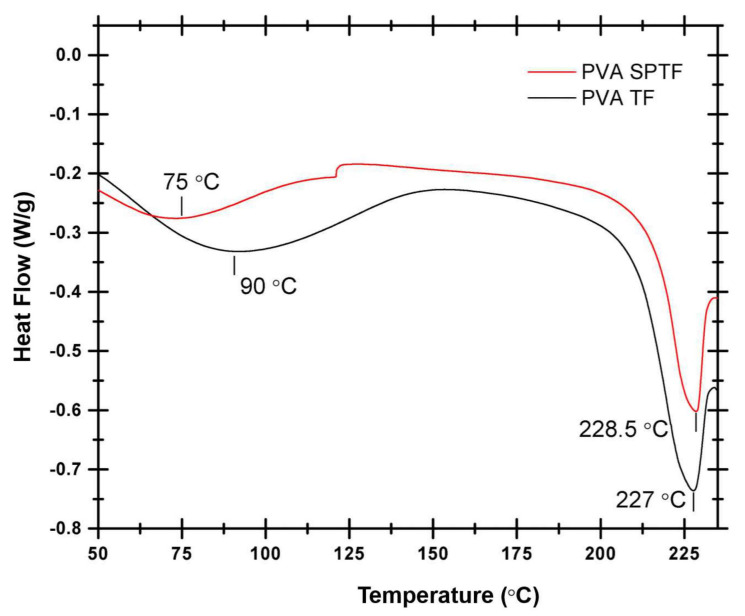
DSC plots of PVA TF and PVA SPTF.

DSC scans are then recorded to determine the glass transition temperature (*T_g_*) and melting point (*T_m_*) of the samples (see Figure 5). Broadly, DSC thermograms show two endothermic peaks. The first peak at around 90 °C depicts the melting of the crystallites of the cross-linked system (PVA/H_2_O) [66]. The second endothermic peak occurring at a relatively higher temperature is attributed to the melting of the PVA [68]. The lower melting temperature pertaining to the cross-linking of PVA/H_2_O decreased from 91 °C for neat PVA to 75 °C for the PVA SPTF. The reduction in the melting temperature pertaining to the cross-linking is attributed to the presence of PAN fibers which hinders the cross-linking in the PVA/H_2_O system. The second endothermic peak in the DSC scan of the PVA TF shows 227 °C as its melting temperature. The endothermic peak at 227 °C recorded for the neat PVA thin film shifts to a slightly higher temperature ∼229 °C in the case of the PVA SPTF. This may indicate a slight increase in the crystallinity of PVA with the addition of PAN in the PVA SPTF. The dynamic mechanical analysis shows higher storage modulus for PVA SPTF as compared to PVA TF (see the Appendix A).

### 3.3. Mechanical Properties of PVA SPTF

#### 3.3.1. Tensile Properties

**Figure 6:**
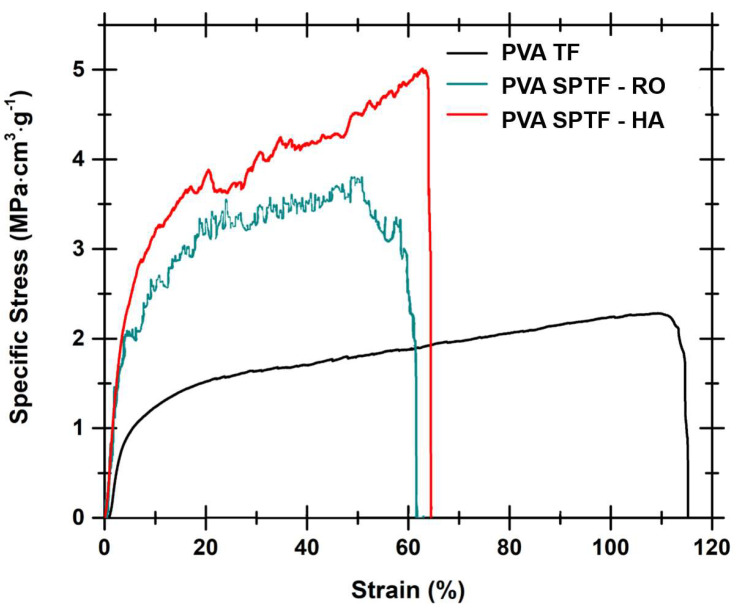
Specific engineering stress vs strain curves for PVA TF, PVA SPTF-HA and PVA SPTF-RO.

The mechanical properties of the reinforced thin films are influenced by the filler type and filler orientation within the matrix. Typically, polymeric thin films reinforced with unidirectionally aligned fibers demonstrate anisotropic mechanical properties, which restricts their applications, while the PVA SPTFs reinforced with a helicoidally arranged PAN fibrous structure show predominately isotropic mechanical properties, making them suitable for varying load conditions. The mechanical deformation behavior of the PVA SPTF reinforced with the helicoidally arranged PAN fibrous structure (PVA SPTF-HA) was evaluated and compared with that of the PVA thin films (PVA TF) and randomly oriented PVA SPTF (PVA SPTF-RO). Figure 6 shows the specific stress versus strain curves for the samples. Specific stress is determined by dividing the measured stress with the density of the sample to account for variation in density between composite as well as neat samples. This methodology is typically followed for fiber reinforced composites to compare them with unreinforced neat samples [48,69].

**Figure 7:**
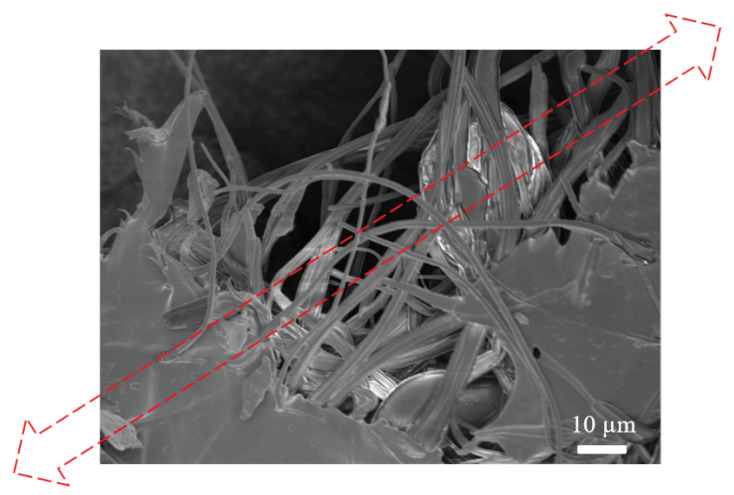
Representative image of the PVA SPTF-HA after the uniaxial tensile test. The figure depicts stretching as well as alignment of the PAN fibers along the tensile loading direction (shown by the red-dashed arrow in the image).

PVA thin film displays large elongation (Figure 6), which is typical of neat thermoplastic polymers [70,71], whereas PVA SPTF-HA exhibits higher specific tensile strength in comparison to the neat PVA thin film and PVA SPTF-RO. The high specific tensile strength of PVA SPTF-HA is attributed to the helicoidally arranged PAN fibers. In PVA SPTF-HA, hydrogen bonding between PAN and PVA can contribute towards transferring load from the stiff PAN fibers to the soft PVA membrane during tensile as well as impact loading. During uniaxial tensile loading in the sample PVA SPTF-HA, the load transfer from PAN to PVA could allow the alignment of the PAN fibers along the tensile stress direction, which can contribute to the increased specific strength of the samples (see Figure 7). Further, the helicoidal arrangement of the PAN fibers, as well as the presence of alternate soft and stiff layer along the thickness of PVA SPTF-HA, generates disparity in modulus along the plane as well as across the planes of the PVA SPTF-HA. This disparity in modulus can hinder the propagation of crack formed during loading, thereby can increase the stress required to propagate crack and cause the failure of PVA SPTF-HA. The ultimate specific tensile strength of the PVA SPTF-HA is determined to be (5 ± 0.3) MPa·cm^3^·g^−1^. This is 52% and 127% higher than the specific tensile strength of PVA SPTF-RO ((3.3 ± 0.4) MPa·cm^3^·g^−1^) and PVA TF ((2.2 ± 0.2 MPa·cm^3^·g^−1^), respectively. Here, ultimate specific tensile strength is equal to the maximum specific tensile stress obtained from the specific stress–strain curve (see Figure 6). In addition, the specific toughness values determined by measuring the area under the specific stress and strain curve is found to be much higher for PVA SPTF-HA ((2.5 ± 0.4) J/g) as compared to PVA SPTF-RO ((1.5 ± 0.4) J/g and PVA TF ((2 ± 0.3) J/g), respectively (see the Table A1 in Appendix A). The low specific toughness for PVA SPTF-RO can be attributed to the randomness of the fibers in the PVA matrix. These random fibers may not align along the tensile stress direction as in the case of PVA SPTF-HA and thus could hinder the elongation of PAN fibers along the tensile stress direction. Further, random fibers give rise to fibers entanglement and/or fiber bundles (see Figure A2 (Appendix A)). These fibers entanglement could give rise to stress concentration zones, which can initiate micro-cracks formation under tensile loading and thereby can result in early failure of PVA SPTF-RO also confined by its low specific toughness values in comparison to both PVA SPTF-HA and PVA TFs. It is interesting to note that the PVA SPTF-HA samples are filled with only ∼1 wt% PAN fibers. Nevertheless, they displayed substantial enhancement of specific tensile strength in comparison to the PVA TF and PVA SPTF-RO samples.

The calculated value of specific tensile strength of the PVA SPTF is found to be comparable (same order of magnitude) to the data available in the literature for different PVA composites [48,56,72,73] and other types of polymer composites [11,72,74,75]. For instance, Ding et al. [72] prepared PVA hydrogels with 2 wt% of PVA, 0.4 wt% borax (PB) and cellulose nanofibers-polypyrrole (CNFs-PPy/PB) and compared it with hydrogel synthesized in the absence of polypyrrole (CNF/PB). They found that the specific compressive stress of CNF-PPy/PB was 18.5 MPa·cm^3^·g^−1^, 1850 times higher than that of CNF/PB (0.01 MPa·cm^3^·g^−1^). Further, silica micrfiber reinforced polybenzoxazine achieved specific tensile strength 90 MPa·cm^3^·g^−1^ for the silica microfiber loading as high as 40% by volume [11]. Wu et al. [74] used thermally expandable microspheres and silicon-containing arylacetylene (SA) resin to fabricate poly(silicon-arylacetylene) syntactic foams. Furthermore, the PSA foam was reinforced with attapulgite (ATT) nanoparticles. The specific compressive strength of the PSA syntactic foam was as high as 25.6 MPa·cm^3^·g^−1^. Zhang et al. [76] obtained low-density and high-performance EMI shielding carbon foam through direct carbonization of the phthalonitrile (PN)-based polymer foam with specific compressive strength of 6.0 MPa·cm^3^·g^−1^. Pilla et al. [75] fabricated and documented the effects of hyperbranched polyesters (HBPs) and nanoclay on the mechanical properties of both solid and microcellular polylactide (PLA). The addition of hyperbranched polyesters (HBPs) and nanoclay increased the specific toughness but resulted in the reduction of specific strength compared with neat PLA samples. The maximum specific strength obtained for PLA samples was 50 MPa·cm^3^·g^−1^.

#### 3.3.2. Impact Resistance

**Figure 8:**
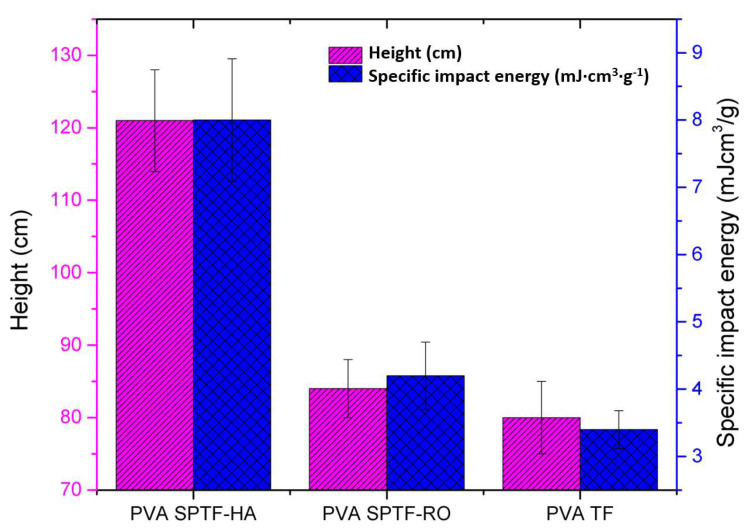
The plot show heights of the free-falling ball, and its corresponding impact energy required to break the glass coverslip protected by the samples. Each bar in the plot depicts the data summarized from the impact test of five individual samples. The error bars represent the range of the data presented in the bar chart.

Impact resistance of the PVA SPTF is determined using free-falling spherical steel ball impact tests [12,48]. Although for smaller samples, we lack the appropriate testing methodology (standards such as ASTM are only documented for bulk or large scale samples) to compare impact performance of the novel materials, we used free-falling ball impact testing [48] methodology to compare at least qualitatively (with other parameters kept constant) and understand the effects of the modifications we have made in terms of the synthesis of the novel materials and thus their microstructural features (such as interfacial adherence between layers as well as between fibers) on the resulting impact performances. In this case, the potential energy of the impacting steel balls is used to test the impact resistance of the samples. The impact energy can be varied either by changing the mass or height of the impacting steel balls [12]. In the first step, the samples are adhered on top of a glass coverslip. Following this, the height and mass of the steel ball required to initiate a macroscopic crack in the glass coverslip that is protected by the samples are measured. For simplicity, the mass (m) of the spherical steel ball used is kept constant at 0.51 g (diameter 4 mm). A bare glass coverslip is used as a control sample. Here, the data are summarized from the impact test of five individual samples. This control sample is seen to break when the steel ball falls from an average height (h) of (20 ± 2) cm. In the case of PVA TF, the glass coverslip is seen to fracture when the steel ball is dropped from an (80 ± 5) cm height (see Figure 8) and for PVA SPTF-RO the glass coverslip fractures at (84 ± 4) cm (see the Table A2 in Appendix A). In contrast, glass coverslip covered with the PVA SPTF-HA fractures when the steel ball is dropped from a (121 ± 7) cm height (see Figure 8). The high impact energy required for the PVA SPTF-HA ((8 ± 0.9) mJ·cm^3^·g^−1^) in comparison to the PVA SPTF-RO ((4.2 ± 0.5) mJ·cm^3^·g^−1^) and PVA TF ((3.4 ± 0.3) mJ·cm^3^·g^−1^) to break the glass coverslip is attributed to both its efficacy and efficiency in dissipating energy through alternatively arranged layers of the soft PVA and relatively stiff PAN fibers in a plane as well as across the plane. Trends from both of these data sets (height when glass coverslip’s fracture is observed and impact energy required) are consistent with our earlier observation with the specific toughness (significantly higher for PVA SPTF-HA compared to other samples), which is also a measure of capacity of the samples to absorb damage/energy during deformation.

This phenomenon can be further explained by assuming that the potential energy of the impacting steel ball was sufficient to deform the first stiff PAN fibrous layer along the thickness of the PVA SPTF-HA. After deforming the PAN fibrous layer, a portion of available potential energy typically dissipates to the adjacent soft PVA matrix material. Therefore, the remaining potential energy may not be enough for deforming the next stiff PAN fibrous layer. Further, these helicoidally arranged PAN fibers intensify the disparity in modulus along the thickness of the PVA SPTF-HA, making it difficult for the deformation induced crack to propagate through it, thus providing high impact resistance in comparison to the PVA thin films as well as PVA SPTF-RO. In the case of PVA SPTF-RO, random PAN fibers could not provide sufficient resistance to the applied potential energy as was the case for PVA SPTF-HA. The applied potential energy is predominately used for the deformation of soft PVA matrix material, which later transfers the energy to the glass slip (protected by PVA SPTF-RO) and resulting in the initiation of cracking in the glass slip at a relatively low applied potential energy.

By virtue of the layer-by-layer approaches of additive manufacturing, the novel fabrication methodology as reported in this present manuscript has shown its broad scope to allow control or modulation in the interfacial adherence between fibers and between layers. Such a capability is important for the design and development of novel materials with highly tunable impact resistances.

## 4. Conclusions

High strength and lightweight PVA SPTF were prepared by embedding helicoidally arranged uniaxially aligned electrospun PAN fibers in PVA through pp-FFES. Tensile and impact tests were performed to verify the specific strength and impact resistance of the PVA SPTF-HAs. The result shows that the PVA SPTF-HAs possess better specific tensile strength and higher resistance to impact compared to the corresponding neat PVA thin films and randomly fiber oriented PVA SPTF. The excellent mechanical properties such as high specific strength and impact resistance of the PVA SPTF-HAs are attributed to its efficient dissipation of energy as well as load transfer during impact and/or uniaxial loading by virtue of its helicoidally arranged PAN fibers network in the PVA thin films (enabled by electrospinning-based processing as an additive manufacturing methodology). Such high specific strength and impact resistant helicoidally arranged fiber reinforced PVA SPTF may have a wide variety of applications ranging from army combat wear and sports gear to novel polycarbonate-based solar photovoltaics (PV) modules.

## Figures and Tables

**Figure A7:**
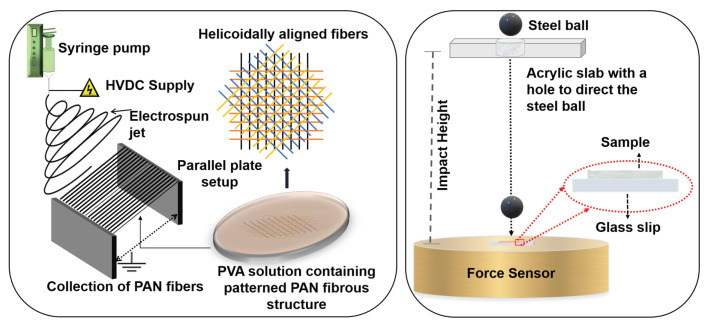
Graphical Abstract.

## Abbreviations

The following abbreviations are used in this manuscript:

pp-FFES	Parallel plate far field electrospinning
TF	Thin films
SPTF	Strong polymer thin films
PAN	Polyacrylonitrile
PVA	polyvinyl alcohol
PVA SPTF-RO	PVA strong polymer thin films with randomly oriented PAN fibers
PVA SPTF-HA	PVA strong polymer thin films with helicoidally arranged PAN fibers

## Appendix A

The calculations were based on cross-sectional area = *t* × *w*, where t is the thickness of the sample, w is the width of the sample, tensile stress = applied load/cross-sectional area, density of the sample = mass/volume and specific tensile stress = tensile stress/density. Ultimate specific tensile strength used for the calculations of specific toughness is equal to maximum specific tensile stress obtained from the specific stress -strain curve and the stain is measured at complete failure.

**Table A1:** Specific tensile properties of the samples PVA TF, PVA SPTF-RO and PVA SPTF-HA.

Sample	Ultimate Specific Tensile Strength	Strain	Specific Toughness	Density
	(MPa·cm^3^·g^−1^)	(%)	(J·g^−1^)	(g·cm^−3^)
PVA TF	2.2 ± 0.2	114 ± 3	2.2 ± 0.3	1.1
PVA SPTF-RO	3.3 ± 0.4	61.4 ± 6	1.5 ± 0.4	1
PVA SPTF-HA	5 ± 0.3	64.4 ± 14	2.5 ± 0.4	0.7

**Table A2:** Specific tensile properties of the samples PVA TF, PVA SPTF-RO and PVA SPTF-HA.

Sample	Impact Height at Which	Specific Impact Energy
	Glass Beneath Sample Breaks	
	(cm)	(mJ·cm^3^·g^−1^)
Coverslip	20 ± 2	-
PVA TF	80 ± 5	3.4 ± 0.3
PVA SPTF-RO	84 ± 4	4.2 ± 0.5
PVA SPTF-HA	120 ± 7	8± 0.9

The impact energy of the glass sample is 1 mJ, for PVA TF is 4 mJ, for PVA SPTF-RO is 4 mJ and for PVA SPTF-HA is 6 mJ.

**Dynamic mechanical analysis (DMA):** The viscoelastic properties of the PVA SPTF and PVA TF are shown in Figure A3. The storage modulus is an indicator of the energy stored during the deformation. The specific viscoelastic properties of the material are determined by dividing the modulus by the density of the samples. The specific storage modulus of the PVA SPTF is determined to be 2650 MPa·cm^3^·g^−1^, while for PVA TF the value is determined to be 1700 MPa·cm^3^·g^−1^. Over the entire temperature range, the specific storage modulus and the loss modulus values of PVA SPTF are higher than PVA TF. For PVA TF, the first tan delta peak is observed at 32 °C corresponding to the α phase relaxation, and there is a second one at 112 °C, corresponding to the β relaxation. The tan delta peaks of the PVA SPTF are seen at 29 °C and 99 °C. The *Tg* of the PVA SPTF reduces in comparison to the PVA TF. These results agree with our DSC results indicating that the presence of PAN fibers hinders in the crosslinking of PVA/H_2_O system.
